# The efficacy of Mycophenolate mofetil for the treatment of Chinese Takayasu’s arteritis

**DOI:** 10.1038/srep38687

**Published:** 2016-12-07

**Authors:** Jing Li, Yunjiao Yang, Jiuliang Zhao, Mengtao Li, Xinping Tian, Xiaofeng Zeng

**Affiliations:** 1 Department of Rheumatology and Clinical Immunology, Peking Union Medical College Hospital (PUMCH), Peking Union Medical College and Chinese Academy of Medical Sciences, Key Laboratory of Rheumatology and Clinical Immunology, Ministry of Education, Beijing, 100032, China

## Abstract

To investigate the therapeutic effect of mycophenolate mofetil(MMF) on Chinese Takayasu’s arteritis(TAK) patients. Thirty consecutive TAK outpatients were prospectively enrolled during 2013 to 2015. MMF combined with glucocorticoid was the primary treatment regimen. If clinical stable disease could not be reached, another traditional immunosuppressive agent could be added. All patients were evaluated and followed up every 3 months and vascular image studies by Doppler ultrasonography were repeated every 6 months. The effectiveness of MMF was defined as:(1) ESR < 20 mm/hr;(2) CRP < 10 mg/L or hs-CRP<3 mg/L;(3) stable or improved in vascular image studies;(4) clinical assessment is stable, improved or in remission;(5) the dosage of glucocorticoid could be tapered to less than 15 mg/day. ESR < 40 mm/hr, CRP < 20 mg/L or hs-CRP < 6 mg/L, but meet the other three criteria is defined as partial effectiveness. MMF alone combined with corticosteroid was effective in 12(40.0%) patients. When MMF combined with methotrexate less than 15 mg/week, the effective rate was 30.0%(9/30), including partial effective in 3 patients. When MMF combined with azathioprine 100–150 mg/day, the effective rate was 10.0%(3/30), including partial effective in 1 patient. Four patients withdrew due to side effects. Two patients failed to show response. The overall effective rate of therapy including MMF in treating TAK is 80%.

Takayasu’s arteritis (TAK) is a rare, chronic, granulomatosis idiopathic systemic vasculitis mainly involves aorta and its major branches^1^. The majority patients are women at child-bearing age^2^. Inflammation of the three layers of the arterial wall and subsequent proliferation results in narrowing and occlusion of the lumen of arteries, sometimes destruction of the elastica and muscularis may result in artery dilation or aneurysm^2^. The presence of inflammatory cell infiltration demonstrated at autopsy suggests the role of cell-mediated autoimmunity in the pathogenesis and morphogenesis of TAK^3^.The major clinical manifestations are caused by the ischemia of the organs supplied by the involved arteries as well as systemic inflammation.

Approximately 50% of TAK patients are sensitive to the treatment of glucocorticoids (GCs)^4^ alone. However, many patients need high maintenance dosage of steroid, which increases the risk of chronic steroid toxicity^5^. Immunosuppressive drugs are usually used to allow reduction of GCs dosage, but only one third of patients show response to immunosuppressive agents^2^. Cyclophosphamide has been considered effective in most glucocorticoid-resistant TAK patients^4^, but its long-term use causes significant toxicity, including infertility, major infections, cystitis, bladder cancer and secondary malignancies^6^, which limit its usage in TAK patients. Other immunosuppressive drugs, including methotrexate (MTX)^5^, azathioprine (AZA)^7^, cyclosporine^8^ and leflunomide^9^, are alternative options in TAK treatment. However, only small sample-size case series of clinical effectiveness were reported in the literature. Biologic drugs, such as anti-tumor necrosis factor and anti-interleukin 6 antibodies, have been tried in the treatment of TAK and have shown promising effect, but the high cost of biologic drugs limits their usage in underdeveloped areas.

Mycophenolate mofetil (MMF) has been reported in a few studies as a useful alternative for TAK treatment^10^,^11^,^12^. This study was designed to investigate the efficacy and tolerability of MMF in Chinese TAK patients.

## Results

### Demographic data, baseline data, and previous medication summary

Thirty TAK patients were treated with MMF in this study. The demographic and baseline data were summarized in Table 1. 90.0% (27/30) patients were female, and the median age at onset was 24.5 (19.8, 32.0) years old. The median disease duration at diagnosis was 11.5 (3.8, 27.0) months, and the median follow-up time was 17.0 (11.0, 28.0) months. Prior to commencing MMF usage, the median disease duration was 12.0 (7.5, 36.0) months (Table 1).

Malaise (63.3%) and headache (53.3%) were the common complaints. Vascular bruit (83.3%) was the most common findings in physical examination in this group of patients, followed by asymmetric blood pressure (80.0%), pulsation (66.7%) and hypertension (56.7%). Elevated C-reaction protein (CRP) and/or high sensitive CRP (hs-CRP) (86.7%) were the most common abnormal laboratory tests, and elevated erythrocyte sediment rate (ESR) was found in 76.7% of patients. Mild anemia was found in 30.0% patients (10/30), in whom the level of hemoglobin was less than 110 g/L (Table 1).

The angiographic classification^13^ at the baseline was shown in Table 1. Twelve patients (40.0%) were classified as type I, seven patients (23.3%) were type IIa, six patients (20.0%) were type V, three patients (10.0%) were type IIb, and two patients (6.7%) were type IV.

Seventeen patients (17/30, 56.7%) received MMF as the first immunosuppressive agent, while the other thirteen patients received MMF as an alternative or additive immunosuppressive drug (ISD) due to uncontrolled disease activity. All patients received glucocorticoids (GCs) at the same time, and the dosage of steroids were tapered during the study period (Table 1).

The mean dosage of GCs when MMF was initiated was 40.0 (14.4, 40.0) mg in 24 patients who showed effectiveness during follow-up, and the mean dosage of GCs on last follow-up in these patients was 10.0 (7.5, 10.0) mg. The duration for dosage of GCs less than 15 mg was 12.0 (9.0, 21.8) months, and the duration of GCs less than 10 mg was 9.0 (3.0, 16.5) months. Three patients among them (3/24, 12.5%) stopped GCs at the last follow-up (Table 1).

### Response towards the treatment of MMF

In this study, 4 patients withdrew MMF due to adverse events. One patient developed skin rash 2 weeks after MMF administration, 2 patients complained of severe gastrointestinal discomfort, and HBV flare occurred in 1 patient. Fourteen patients received MMF and steroid combined with other immunosuppressive agent when their diseases was still active. The follow-up time after initiation of MMF in MMF combined with GCs group (median 24.5 months) was longer than that in patients with MMF combined with another immunosuppressive agent treatment group (median 11.0 months) (Table 1). Excluding 4 patients withdrawal due to side effect of MMF, 26 patients were followed up for more than 6 months.

40.0% (12/30) patients in the MMF combined with GCs group could reach and maintain stable disease. With adding MTX less than 15 mg/week or AZA 100–150 mg/day, the dosage of steroid could be tapered to less than 15 mg/day in 12 patients (40.0%). Two patients failed to reach stable disease after being treated with combination of several ISDs including MMF (Table 2). Thus the effective rate of MMF alone, or combined with methotrexate or azathioprine, with low-dosage GCs in most patients, was effective in the treatment of TAK, and the overall effective rate was 80.0%.

### Comparisons of clinical manifestations, laboratory findings, and angiographic presentations

We compared the clinical features and angiographic types between patients who responded to MMF combined with GCs (n = 12), and MMF combined with other ISD and GCs (n = 12) (Table 1). There was no significant difference in clinical manifestations between these groups. But the disease duration at diagnosis (5.0 vs 18.5 months, *p* = 0.014) and prior to initiation of MMF (10.0 vs 18.5 months, *p* = 0.024) were shorter in MMF combined with GCs group (n = 12) than in MMF combined with other ISD and GCs group (n = 12). And the follow-up time after initiating MMF was longer in MMF combined with GCs group (27.5 vs 11.0 months, *p* = 0.005).

There was no significant difference in the dosage of GCs used when MMF treatment was initiated and on last follow-up between MMF combined with GCs group (n = 12) and MMF combined with other ISD and GCs group (n = 12). But the duration for the dosage of GCs tapered to less than 15 mg (21.5 vs 10.0 months, *p* = 0.024) and 10 mg (14.0 vs 6.0 months, *p* = 0.033) was longer in the former group.

### Comparisons of involved vessels distribution

For the vessels involved in these 30 TAK patients, carotid artery and subclavian artery were involved in 22 patients (73.3%). The distribution of vessels involved between the MMF combined with GCs group (n = 12) and the combined treatment including MMF group (n = 12) was not significantly different (Table 3).

## Discussion

TAK is a chronic progressive large vessel vasculitis^3^. Autopsy in Japanese TAK patients disclosed various lesions, including acute exudative inflammation, chronic non-specific productive inflammation, and granulomatous inflammation in the media and adventitia through the casa vasorum. The new active lesions were often observed near the old fibrotic ones, which suggested that TAK might be a progressive disease^3^.The disease activity assessment and treatment are still challenging. Due to its relatively low prevalence, there has been no controlled trial in TAK. Diverse immunosuppressive drugs had been examined in small sample-size case series or case reports, including CTX, MTX, AZA, cyclosporine and leflunomide^4^,^5^,^7^,^8^,^9^. MMF has been shown to be effective as an alternative steroid-sparing agent in three case series, including three, ten and twenty-one TAK patients, respectively^10^,^11^,^12^.

As an inosine monophosphate dehydrogenase inhibitor, MMF could interfere DNA synthesis by inhibiting guanine nucleotide synthesis. Since lymphocytes depend primarily on de novo purine synthesis while neutrophils utilize salvage pathway, MMF inhibits proliferation of lymphocytes specifically^14^. Several large clinical trials have demonstrated the role of MMF in preventing acute transplant rejection^15^. And in experimental models, MMF prevents arterial smooth muscle-cell proliferation and proliferative arteriopathy, which were common mechanisms involved in chronic transplant rejection^16^,^17^. Based on these mechanisms, MMF has been suggested to inhibit the vasculitis mediated by lymphocytes, such as TAK. In fact, MMF had been demonstrated to be effective in various systemic vasculitides, including ANCA-associated vasculitis^18^ and lupus nephritis^19^.

In this study, we have demonstrated that MMF may be effective in controlling disease activity and vessel damage progression, either combined with low-dosage GCs or combined with MTX or AZA, with the total effective rate up to 80.0%. But in the combination of immunosuppressive agents (the combination regimen) and MMF group, the follow-up time after administration of MMF (median 11.0 months) is shorter than in MMF plus GC group (median 27.5 months) (*p* = 0.005). And four patients in the combination regimen group is partial effective, demonstrating no progression of vascular lesions but with elevated level of ESR/CRP, which was lower than two times of the upper normal limit.

When the age at disease onset, history of medication usage, clinical symptoms, physical examination findings, laboratory tests, angiographic classification, and the distribution of involved vessels were compared between patients based on the efficacy and regimen used, no significant difference could be found. It suggests that MMF could be indicated in any TAK patients with active disease. In this study, in patients of the MMF plus GCs group (n = 12), the disease duration at diagnosis (median 5.0 months) and prior to commencing of MMF (median 10.0 months) were significantly shorter than in the combined regimen group (n = 12).The immunosuppressive effect of MMF in autoimmune disease was speculated by inhibiting lymphocyte-mediated immune process. Therefore, we may speculated that early administration of MMF in TAK patients may be beneficial in slowing down or preventing the disease progression.

For the side effects of MMF, Shinjo *et al*.^11^ had reported headache as the only adverse events, and Goel *et al*.^10^ had reported skin rash and sepsis. In this study, two patients experienced unbearable gastrointestinal discomfort, one had pruritic rashes, and one patient suffered a flare of previous HBV infection 6 weeks after administration of MMF. Discontinuation of MMF and addition of entecavir suppressed the replication of HBV, and later addition of MTX and AZA did not induce replication of HBV within 5 months follow-up. No cytopenia, liver enzyme elevation, or renal dysfunction was observed in our patients.

Doppler ultrasonic imaging was repeated to measure and compare the thickness of the vessel walls and lumen diameter of vessels by one technician specialized in vascular ultrasonic examinations. As there is no widely accepted image modality to evaluate the disease activity of angiographic lesions, various image modalities have been used in different clinical trials. Magnetic resonance angiography (MRA)^20^ and positron emission tomography (PET)^21^,^22^ are reported to be sensitive in evaluating vessel inflammatory changes, but their role in daily practice and disease activity follow-up has not been well established. The high cost of MRA and PER-CT limits its usage in Chinese TAK patients.

The major limitation of this study is the relatively small sample size, though this is the largest cohort reported in the literature according to our knowledge. Another limitation is the relatively short follow-up time for a chronic disease as TAK. In this study, the median follow-up time was 17.0 months. As TAK is a chronic disease, so 17 months follow-up duration might not be long enough to provide solid evidence to assess disease progression. Perhaps further follow-up of these patients is needed to confirm the effectiveness of these regimens in delaying or halted the progress of the disease. Therefore, large, long-term follow up studies are needed to verify the effectiveness of MMF in TAK treatment.

In summary, this study has demonstrated that, MMF, and MMF combined with MTX or AZA, when combined with daily low dose GCs, are tolerable and effective in 80.0% of TAK patients. Neither angiographic progression shown on Doppler ultrasonography nor toxicity of vital organ/system has been observed during follow-up. Therefore, we may conclude that MMF may be an effective alternative for disease control and tapering of steroid in the treatment of TAK. It offers a good choice for active TAK patients.

## Patients and Methods

### Patients

Thirty consecutive TAK patients were treated with oral MMF, and were followed-up at the outpatient Vasculitis Clinic, Department of Rheumatology of Peking Union Medical College Hospital (PUMCH), a Chinese nationwide referral center during January 2013 to September 2015. All patients fulfilled the classification criteria of modified 1990 American College of Rheumatology (ACR) criteria^23^. Patients who had one of these criteria were excluded: (1) intolerant or failed to response to MMF in the past history of treatment; (2) thiopurine methyl transferase (TPMT) gene test showed mutations; (3) un-controlled diabetes or hypertension before entry; (4) active digestive tract bleeding 3 months before entry; (5) severe liver and kidney dysfunction, and damage defined as the alanine aminotransferase (ALT) or aspartate aminotransferase (AST) level were 3 times above the upper limits of the normal range and the serum creatinine level was 1.5 times above the upper limits; (6) patients with active infection, including bacteria, virus, fungi and mycobacteria. All patients were in active disease based on the National Institutes of Health (NIH) criteria^2^, defined as new-onset or worsening in at least two of the following: (1) systemic features, such as fever, musculoskeletal involvement (excluded other causes); (2) manifestations of ischemia or inflammation in vessels, such as claudication, pulse deficit, bruits, vascular pain (carotidynia), asymmetric blood pressure in upper and/or lower limbs; (3) elevated ESR and/or CRP protein levels, excluding the existence of active infection; (4) typical angiographic features.

These patients received oral MMF, 1.5 to 2 gram per day, and were followed up for a minimum period of 6 months. All patients were evaluated and followed up every 3 months and vascular image studies were repeated every 6 months. Clinical manifestations of these patients were reviewed, including symptoms, physical findings, laboratory findings, and vascular involvement. Their medication records and response to MMF were categorially summarized. The effectiveness of MMF was defined as: (1) ESR < 20 mm/hr after treatment; (2) CRP < 10 mg/L or hs-CRP < 3 mg/L after treatment; (3) stable or improved in vascular image studies; (4) clinical assessment of disease activity is stable, improved or in remission after treatment; (5) the dosage of corticosteroid could be tapered to less than 15 mg/day (equivalent dosage of prednisone). ESR < 40 mm/hr, CRP < 20 mg/L or hs-CRP < 6 mg/L, but fulfilled for the other three criteria was defined as partial effective. The primary end-point of this study is the percentage of patients with full and partial effectiveness.

### Methods

These patients were prospectively enrolled and followed-up every 3 months. The demographic data, disease duration, clinical symptoms, physical findings, disease activity status, laboratory findings, angiographic presentations, and treatment modalities, including medication regimens and adverse drug reactions, were collected at baseline and at each follow-up visit. During follow up, we repeated the examination of involved vessels by vascular Doppler ultrasonic imaging, measuring and comparing the thickness of the vessel walls and lumen diameter of vessels.

The study protocol was approved by Institutional Review Board of PUMCH, and all methods were performed in accordance with the relevant guidelines and regulations. Written informed consent was obtained for each participant. This study was registered in Chinese Clinical Trial Registry (http://www.chictr.org.cn) on Jul 25, 2016. The registration number is ChiCTR-OPC-16008907.

### Statistical analysis

Due to the small sample size of this study, the variables were not distributed in a normal pattern. We described the numerical variables as median (quartiles), and the categorical variables as number (percentage). Comparisons between groups were made using Mann-Whitney U test for numerical data, and Chi-square tests for categorical data. Fisher’s exact tests were performed when the expected frequencies were less than 5. A two-sided *P*-value less than 0.05 was considered to be statistically significant. Analysis was performed with the SPSS software (version 19.0).

## Additional Information

**How to cite this article**: Li, J. *et al*. The efficacy of Mycophenolate mofetil for the treatment of Chinese Takayasu’s arteritis. *Sci. Rep.* **6**, 38687; doi: 10.1038/srep38687 (2016).

**Publisher's note:** Springer Nature remains neutral with regard to jurisdictional claims in published maps and institutional affiliations.

## Figures and Tables

**Table 1:** Demographic data, major characteristics of 30 TAK patients, with comparisons between treatment including MMF and other immunosuppressive drugs.

	Total *N (%*)	MMF ± GCS *N (%*)	MMF ± GCS + other ISD N (%)	*P*-value	MMF ± GCS effective *N (%*)	MMF ± GCS + other ISD effective *N (%*)	*P*-value	MMF therapy effective *N (%*)
*N*	30	16	14		12	12		24
Gender								
Female	27 (90.0)	13 (81.3)	14 (100.0)	0.23^F^	10 (83.3)	12 (100)	0.48^F^	22 (91.7)
Male	3 (10.0)	3 (18.3)	0		2 (16.7)	0 (0)		2 (8.3)
Age at onset (years)	24.5 (19.8, 32.0)*	28.5 (23.0, 36.8)*	23.5 (17.8, 28.3)*	0.08	28.5 (23.0, 35.3)*	24.0 (18.5, 28.8)*	0.11	25.5 (20.0, 31.5)*
Duration of disease at diagnosis (months)	11.5 (3.8, 27.0)*	10.0 (3.0, 22.5)*	14.0 (5.5, 36.0)*	0.19	5.0 (2.3, 10.8)*	18.5 (7.5, 36.0)*	0.014^$^	10.5 (3.3, 24.0)*
Duration of disease prior to commence of MMF treatment (months)	12.0 (7.5, 36.0)*	10.5 (6.5, 33.0)*	18.5 (10.5, 37.0)*	0.28	10.0 (5.3, 18.5)*	18.5 (12.0, 39.0)*	0.024^$^	12.0 (6.5, 33.0)*
Follow-up time (months)	17.0 (11.0, 28.0)*	25.5 (14.3, 31.0)*	15.5 (11.0, 18.3)*	0.09	27.5 (17.0, 32.8)*	14.5 (11.0, 17.0)*	0.045^$^	17.0 (11.5, 31.0)*
Follow-up time after commence of MMF treatment (months)	14.5 (9.0, 26.3)*	24.5 (9.5, 28.8)*	11.0 (8.8, 16.3)*	0.043^$^	27.5 (16.3, 30.5)*	11.0 (9.0, 16.8)*	0.005^$^	16.5 (9.0, 27.8)*
Previous usage of ISD								
No ISD except GCS	17 (56.7)	10 (62.5)	7 (50.0)	0.48	7 (58.3)	6 (50.0)	0.68	13 (54.2)
One ISD besides GCS	5 (16.7)	3 (18.8)	2 (14.3)	1.00^F^	2 (16.7)	2 (16.7)	1.00^F^	4 (16.7)
More than one type of ISD besides GCS	8 (26.7)	3 (18.8)	5 (35.7)	0.42^F^	3 (25.0)	4 (33.3)	1.00^F^	7 (29.2)
Mean dosage of GCS on commence of MMF treatment (mg)	NS	NS	NS	NS	40.0 (10.6, 40.0)*	40.0 (22.5, 47.5)*	0.29	40.0 (14.4, 40.0)*
Mean dosage of GCS on last follow-up (mg)	NS	NS	NS	NS	8.8 (1.9, 10.0)*	10.0 (10.0, 11.9)*	0.13	10.0 (7.5, 10.0)*
Duration for dosage of GCS no more than 15mg (months)	NS	NS	NS	NS	21.5 (10.0, 24.8)*	10.0 (6.8, 12.8)*	0.024^$^	12.0 (9.0, 21.8)*
Duration for dosage of GCS no more than 10mg (months)	NS	NS	NS	NS	14.0 (4.5, 22.5)*	6.0 (0.8, 9.0)*	0.033^$^	9.0 (3.0, 16.5)*
Ceased the usage of GCS	NS	NS	NS	NS	3	0	0.22^F^	3
Constitutional findings								
Fever	12 (40.0)	NS	NS	NS	5 (41.7)	3 (25.0)	0.67^F^	8 (33.3)
Malaise	19 (63.3)	NS	NS	NS	6 (50.0)	7 (58.3)	0.68	13 (54.2)
Joint pain	7 (23.3)	NS	NS	NS	3 (25.0)	4 (33.3)	1.00^F^	7 (29.2)
Weight loss	8 (26.7)	NS	NS	NS	5 (41.7)	1 (8.3)	0.16^F^	6 (25.0)
Vascular findings								
Pulse deficit	20 (66.7)	NS	NS	NS	10 (83.3)	7 (58.3)	0.37^F^	17 (70.8)
Claudication	12 (40.0)	NS	NS	NS	3 (25.0)	5 (41.7)	0.67^F^	8 (33.3)
Vascular bruit	25 (83.3)	NS	NS	NS	8 (66.7)	12 (100.0)	0.09^F^	20 (83.3)
Hypertension	17 (56.7)	NS	NS	NS	5 (41.7)	8 (66.7)	0.22	13 (54.2)
Asymmetric blood pressure	24 (80.0)	NS	NS	NS	10 (83.3)	9 (75.0)	1.00^F^	19 (79.2)
Cardiac findings								
Angina	1 (3.3)	NS	NS	NS	0 (0)	0 (0)	NA	0 (0)
Congestive heart failure	1 (3.3)	NS	NS	NS	0 (0)	1 (8.3)	1.00^F^	1 (4.2)
Aortic insufficiency	8 (26.7)	NS	NS	NS	3 (25.0)	3 (25.0)	1.00^F^	6 (25.0)
Neurologic findings								
Headache	16 (53.3)	NS	NS	NS	4 (33.3)	7 (58.3)	0.22	11 (45.8)
Visual disturbance	5 (16.7)	NS	NS	NS	2 (16,7)	3 (25.0)	1.00^F^	5 (20.8)
Diplopia	1 (3.3)	NS	NS	NS	0 (0)	1 (8.3)	1.00^F^	1 (4.2)
Retinopathy	2 (6.7)	NS	NS	NS	1 (8.3)	1 (8.3)	1.00^F^	2 (8.3)
Laboratory findings								
Elevated ESR	23 (76.7)	NS	NS	NS	7 (58.3)	11 (91.7)	0.16^F^	18 (75.0)
Elevated CRP	26 (86.7)	NS	NS	NS	9 (75.0)	12 (100.0)	0.22^F^	21 (87.5)
Anemia (Hb < 110g/L)	9 (30.0)	NS	NS	NS	1 (8.3)	5 (41.7)	0.16^F^	6 (25.0)
Numano’s angiographic classification								
Type I	12 (40.0)	NS	NS	NS	6 (50.0)	4 (33.3)	0.41	10 (41.7)
Type IIa	7 (23.3)	NS	NS	NS	2 (16.7)	4 (33.3)	0.64^F^	6 (25.0)
Type IIb	3 (10.0)	NS	NS	NS	0 (0)	1 (8.3)	1.00^F^	1 (4.2)
Type III	0 (0)	NS	NS	NS	0 (0)	0 (0)	NA	0 (0)
Type IV	2 (6.7)	NS	NS	NS	1 (8.3)	0 (0)	1.00^F^	1 (4.2)
Type V	6 (20.0)	NS	NS	NS	3 (25.0)	3 (25.0)	1.00^F^	6 (25.0)

TAK: Takayasu’s arteritis, GCS: glucocorticosteroid, ISD: immunosuppressive drugs, ESR: erythrocyte sediment rate, CRP: C-reactive protein, NS: not statistics.

*Median (quantiles); ^F^Fisher’s exact test.

^$^*P* < 0.05. NA, not available for test.

**Table 2:** Response of 30 TAK patients towards the therapy including MMF.

Treatment and effectiveness	Numbers of Patients	%
MMF alone		
effective	12	40.0
ceased usage of MMF		
side effect of gastrointestinal tract	2	6.7
skin rash	1	3.3
HBV flare	1	3.3
MMF combined with MTX		
effective	6	20.0
partial effective	3	10.0
MMF combined with AZA		
effective	2	6.7
partial effective	1	3.3
Refractory	2	6.7
Total	30	

TAK: Takayasu’s arteritis, MMF: mycophenolate mofetil, AZA: azathioprine, MTX: methotrexate.

**Table 3:** The manifestations of vessels involved in 31 Takayasu’s arteritis patients.

Vessels manifestations	Total *N (%*)	MMF±GCS effective *N (%*)	MMF±GCS+other ISD effective *N (%*)	*P*-value^F^
*N*	30	12	12	
Ascending aorta	2 (6.7)	0 (0)	2 (16.7)	0.48
Stenosis	1 (3.3)	0 (0)	1 (8.3)	1.00
Aneurysm	1 (3.3)	0 (0)	1 (8.3)	1.00
Thoracic aorta				
Stenosis	3 (10.0)	1 (8.3)	1 (8.3)	1.00
Abdominal aorta	2 (6.7)	1 (8.3)	1 (8.3)	1.00
Stenosis	1 (3.3)	1 (8.3)	0 (0)	1.00
Aneurysm	1 (3.3)	0 (0)	1 (8.3)	1.00
Carotid artery	22 (73.3)	8 (66.7)	12 (100.0)	0.09
Stenosis	19 (63.3)	7 (58.3)	9 (75.0)	0.67
Occlusion	5 (16.7)	1 (8.3)	4 (33.3)	0.32
Subclavian artery	22 (73.3)	8 (66.7)	11 (91.7)	0.32
Stenosis	20 (66.7)	7 (58.3)	10 (83.3)	0.37
Occlusion	7 (23.3)	4 (33.3)	2 (16.7)	0.64
Aneurysm	1 (3.3)	0 (0)	1 (8.3)	1.00
Vertebral artery	6 (20.0)	5 (41.7)	1 (8.3)	0.16
Stenosis	5 (16.7)	4 (33.3)	1 (8.3)	0.32
Occlusion	1 (3.3)	1 (8.3)	0 (0)	1.00
Mesenteric artery	3 (10.0)	2 (16.7)	1 (8.3)	1.00
Stenosis	2 (6.7)	1 (8.3)	1 (8.3)	1.00
Occlusion	1 (3.3)	1 (8.3)	0 (0)	1.00
Renal artery				
Stenosis	4 (13.3)	1 (8.3)	2 (16.7)	1.00
Iliacofemoral artery				
Stenosis	1 (3.3)	0 (0)	0 (0)	NA
Pulmonary artery				
Stenosis	1 (3.3)	0 (0)	0 (0)	NA
Coronary artery				
Stenosis	1 (3.3)	0 (0)	0 (0)	NA

^F^Fisher’s exact test. NA, not available for test.
